# Health and Wellness Outcomes of Intimate Partner Violence Support Workers: A Narrative Review

**DOI:** 10.1177/15248380241231604

**Published:** 2024-02-14

**Authors:** Tara Lundy, Joanne Crawford

**Affiliations:** 1 Brock University, St. Catharines, ON, Canada

**Keywords:** domestic violence, violence exposure, vicarious trauma, mental health and violence

## Abstract

Workers who support survivors of intimate partner violence (IPV) witness some of the most traumatic acts of violence in their everyday work life. These experiences may cause distress that has implications for health and their ability to cope. This narrative literature review sought to explore what is known about the health, wellness, and coping strategies of IPV workers in diverse settings. A comprehensive academic literature search of five databases for peer-reviewed journal articles, available in English, published between January 2000 and October 2023 was conducted. A total of 34 articles on workers’ experiences in relation to health, wellness, and coping strategies were included in the review. Thematic analysis generated the following themes: (a) diversity of IPV workplace settings and roles; (b) meaningful aspects of IPV support work including purpose and fulfillment, compassion satisfaction, rewarding and valuable work; (c) adverse experiences such as psychological distress and physiological discomfort, interpersonal social challenges, environment and organizational challenges, burnout, compassion fatigue and secondary trauma; and (d) coping strategies that considered coping behaviors and self-care, workplace support and accommodation, and meaningful sacrifice and adaptation. While the review provided important insights regarding the meaningful aspects of IPV support work and coping strategies, the adverse experiences of supporting survivors significantly dominated the literature. Unfortunately, the majority of studies did not clarify the context of workplaces, and this represents a gap in understanding workers’ experiences. Future research is needed to understand context-related experiences of IPV support workers in relation to health and coping. The current review provides unique insights on diverse IPV support work settings and roles, work-related issues that may influence workers’ wellness, and rewarding aspects of IPV support work.

## Introduction

Intimate partner violence (IPV) is a worldwide public health problem that is defined as “behaviour by an intimate partner or ex-partner that causes physical, sexual or psychological harm, including physical aggression, sexual coercion, psychological abuse, and controlling behaviours” (World Health Organization [WHO], 2021, para. 3). It is associated with several negative acute and chronic health outcomes for survivors such as physical injuries and mental health issues, and engagement in unhealthy coping behaviors (Centers for Disease Control and Prevention [CDC], 2020; WHO, 2012, 2021). Women disproportionally experience IPV; WHO (2021) estimates that globally, one in three or 30% of women have been subjected to IPV in their lifetime and 38% of female homicides worldwide are committed by intimate partners. Moreover, marginalized populations, such as those who may be discriminated against based on gender identity, disability, race, ethnicity, sexual orientation, or migrant status, have an increased risk of abuse (Government of Canada, 2022; Statistics Canada, 2021).

An individual’s working life conditions is a key social determinant of health (WHO, 2023). Workers who support survivors of IPV play an essential role in preventing harm and promoting survivors’ health, healing, and resilience. They face traumatic acts of violence in their everyday work life, which may subsequently impact their health and wellness (Rossiter et al., 2020; Wachter et al., 2020). Holistic health is multidimensional and extends beyond physical health (Global Wellness Institute, n.d.). Wellness is the active process that incorporates the physical, mental, emotional, spiritual, social, and environmental dimensions to achieve holistic health (Global Wellness Institute, n.d.). IPV support workers are employed in various workplace settings where their role, length of exposure and relationships with survivors, and structural workplace conditions may differ. Namely, they may be employed in government sectors with consistent funding and mandates in which they provide short-term support in acute crises (Government of Canada, 2015; Rossiter et al., 2020). In contrast, they may be employed in for-profit private settings or non-profit community-based organizations or agencies that are independent, not publicly funded, and not bound by government policy or practice (Government of Canada, 2015). In the later circumstance, support workers can often be the first contact for survivors after experiencing abuse, but also provide long-term emotional, informational, and appraisal support for survivors in their healing journey (Bell, 2003; Government of Canada, 2015; Kulkarni et al., 2013; Rossiter et al., 2020). Research on the health and wellness of IPV support workers is essential given their role in providing vital services and compassionate care during a survivor’s deeply challenging time. A report on vicarious trauma and IPV suggests that anti-violence workers may experience difficulty managing emotions, relationship and connection challenges with friends or family, or a sense of hopelessness and loss of meaning in life (Tabibi et al., 2021). Ongoing exposure to the suffering or trauma experienced by survivors may cause distress, chronic stress, unpleasant physiological symptoms, or interpersonal conflicts which may challenge them personally to cope and provide adequate support to survivors. The purpose of this narrative review was to understand and answer the question, “what is known about IPV workers’ experiences in relation to health, wellness, and coping strategies?” Two review studies (Brend et al., 2020; Sutton et al., 2021) that similarly explored this topic either focused on aspects of burnout and/or did not clarify the context of workplaces. The current review provides a better understanding about the wellness-related issues that IPV support workers may experience, as well as unique insights on diverse IPV support work settings and roles. Further, the review focuses not only on work-related distress but also rewarding aspects of IPV support work, such as compassion satisfaction, and a sense of purpose. This research will be of significance to IPV services, organizations, and others working in the field of IPV, as it can provide insights on the experiences related to IPV support workers’ health, wellness, and coping strategies in this field.

## Method

### Narrative Literature Review

Narrative reviews are useful for describing, analyzing, and synthesizing ideas and concepts from a range of literature on a specific topic (Kiteley & Stogdon, 2014). Green et al.’s (2006) approach for conducting a narrative interview review and the Preferred Reporting Items for Systematic Reviews and Meta-Analyses (PRISMA) (Page et al., 2021) checklist was used as a guide to maintain consistency and achieve a comprehensive review. The process entailed: (a) performing a preliminary search of the literature; (b) refining a topic and formulating a research question; (c) developing and refining a search strategy; (d) reviewing relevant literature from multiple databases; (e) selecting studies based on inclusion and exclusion criteria; and (f) describing, critically analyzing, and narratively synthesizing the literature findings.

#### Search Strategy

Consistent with narrative review methods (Green et al., 2006), a comprehensive search using a combination of terms were performed in each of the following databases for the academic literature review: CINAHL, PsycINFO, Embase, OVID Medline and ProQuest Sociology Collection. These were the most relevant databases that covered health, behavioral social sciences, nursing, mental health, and biomedical research. Controlled vocabularies of each database were included, and a combination of the following key search terms yielded a total of 1,338 citation returns after removal of 82 duplicates: “intimate partner violence” or “intimate partner violence and abuse,” and “work*” or “social worker*” or “service work*” or “counsellor*” or “therapist*” or “social service worker*” or “community work*” or “community anti-violence work*” or “non-statutory” or “intimate partner violence advocate*” or “domestic violence advocate*” or “intimate partner violence agency” or “domestic violence agency,” and “health” or “wellness” or “vicarious trauma*” or “secondary traumatic stress” or “burnout” or “compassion fatigue” or “occupational stress” or “satisfaction” or “self-worth” or “vicarious resilience” or “positive coping” or “healthy coping” or “protective factor*” or “post-traumatic growth” and “not child*.”

#### Selection Process

The initial purpose of the review was to understand what was known about the experiences of IPV support workers who were employed in non-profit, community-based settings in relation to health, wellness, and coping strategies. However, the search needed to be broadened because of the limited literature on the experiences of IPV support workers in for-profit private and non-profit community-based settings, and because of the lack of clarity reported by authors on the context and workplace settings of participants. Therefore, the inclusion criteria was broadened to include the experiences of IPV support workers from diverse workplace settings, in order to gain insight on work experiences and its influence on health and wellness overall.

#### Inclusion Criteria

Published studies were included in the review if they were: (a) reported in English; (b) peer-reviewed academic journal articles; (c) published from January 2000 to October 2023; and (d) involved employed IPV support workers’ experiences in relation to health, wellness, and coping strategies. The time period was selected to account for when data began to emerge in the literature.

#### Exclusion Criteria

Studies that focused on workers who: (a) exclusively supported child survivors of violence and abuse, or (b) exclusively supported perpetrators of violence were excluded from the review as their work experiences may be different. Five studies in this review included others in the sample such as students, volunteers, workers who supported survivors of family violence, sexual assault, and perpetrators of violence. From each of these studies, the current review only reported on relevant findings related to employed workers and did not encompass volunteer or charity workers.

#### Data Management and Synthesis

**Figure 1.**
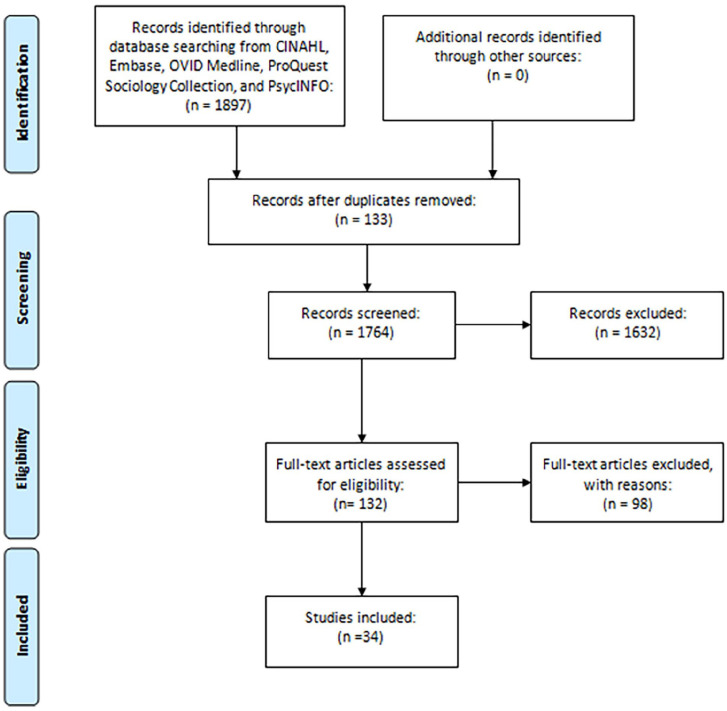
Narrative literature review process.

The lead author conducted each stage of the review, with review by the second author. The academic literature search revealed a total of 1,897 citations, which were exported to EndNote, and 1,764 citations remained after 133 duplicates were removed. After an initial title and abstract screen, 132 full-text articles were relevant and assessed for inclusion; reason for exclusion were documented. Data was charted utilizing an excel spread sheet and included study author(s), date of publication, country of origin, purpose and aim, target population, research method, and findings. Narrative synthesis was guided by Braun and Clarke’s (2012) thematic analysis (see Figure 1). The process involved coding data descriptively, and generating common themes across studies; both authors met to discuss concepts and reach consensus on final themes.

## Findings

The 34 studies included in this review were conducted in the: United States (*n* = 20), Africa (*n* = 3), Israel (*n* = 3), Canada (*n* = 2), Spain (*n* = 2), Australia (*n* = 1), Malaysia (*n* = 1), United Kingdom (*n* = 1), and a scoping review included papers from diverse countries. Three research studies used a mix-methods design, 10 employed quantitative designs, 19 were qualitative inquiries, and two were scoping or systematic reviews (See Table 1). Themes were generated based on common and interconnected patterns of IPV support work across literature. The following sections present four overarching themes including the diversity of workplace settings and roles, meaningful and adverse experiences, and coping strategies, all of which relate to health and wellness of IPV support workers.

**Table 1.** Overview of Key Findings in Included Studies.

Author	Methods	Population	Work Setting	Key Findings
AbiNader et al. (2020)	Qualitative interviews	*N* = 9. IPV advocates who served clients of IPV homicide.	(a) Government-based: State agencies.	(a) Experiences of intense, emotional reactions after homicide of a client. (b) In-person co-worker support, debriefing, acknowledging vicarious trauma, and flexible agency policies were important in managing isolating feelings, distress, promoting resilience, and processing homicide.
Alani and Stroink (2015)	Qualitative interviews	*N* = 7. Female sexual and domestic violence service providers who work in a range of health and service professions and hold mental health and counseling roles to serve female survivors of IPV.	(a) Unknown: Participants worked in a range of organizations.	(a) Workers expressed a sense of purpose from supporting survivors. (b) They experienced a lack of control to effectively support clients due to limiting organization policies, scarce community resources, short-term funding, and inaccessible information for clients. (c) They expressed frustration related to the lack of accountability on men for their violent behavior. (d) Helpful self-care strategies included adequate vacation time, regular social support, eating well, physical activity, spirituality connections.
Bell (2003)	Qualitative interviews	*N* = 30. Counselors who serve female victims of domestic violence.	(a) Government-based: Law enforcement agency, courts. (b) For-profit private service: Private therapy practice. (c) Unknown: Shelter, hot line.	(a) Counselors felt their work was rewarding and satisfying. (b) They experienced symptoms of secondary traumatic stress. (c) They experienced social disruptions in their intimate relationships. (d) Having a sense of competence about coping and drawing upon positive role models helped with work-related stress.
Bemiller and Williams (2011)	Quantitative survey	*N* = 194. Domestic violence/sexual assault shelter advocates for domestic/sexual assault victims.	(a) Unknown: Domestic and sexual assault shelters.	(a) Workers experienced moderate levels of burnout, job stress, and had few external rewards. (b) They viewed themselves as the “good soldier” to alleviate burnout.
Ben-Porat (2015)	Quantitative survey	*N* = 214. Domestic violence and non-domestic violence social service therapists.	(a) Unknown: Violence prevention centers, battered women shelters, social service departments.	(a) High levels of secondary trauma and heavy workloads resulted in a decline of vicarious post-traumatic growth. (b) Domestic violence therapists reported lower levels of vicarious post-traumatic growth than non-domestic violence therapists.
Ben-Porat (2017)	Quantitative survey	*N* = 214. Social workers who serve survivors of family violence, women survivors of violence.	(a) Government-based: Social service bureaus. (b) Unknown: Centers for prevention of family violence, shelters for victims of violence against women.	(a) Workers reported symptoms characterized by secondary trauma that undermined their confidence to effectively support their clients. (b) Higher levels of managerial support significantly contributed to workers’ sense of role competence.
Brend and MacIntosh (2021)	Qualitative interviews	*N* = 5. Social Workers who serve people impacted by IPV, including survivors and perpetrators of IPV.	(a) Non-profit community service.	Workplace social support assisted in moderating secondary traumatic stress.
Briones-Vozmediano et al. (2014)	Qualitative interviews	*N* = 43. Professionals (social workers, psychologists, intercultural mediators, judges, lawyers, police officers, health professionals) who serve immigrant women experiencing IPV.	(a) Government-based: Public institutions. (b) Unknown: Non-governmental organizations, specialized services.	Staff shortages, increased workloads, funding cutbacks, and reduced salaries hampered workers’ ability to provide effective services and made them feel underappreciated.
Brown et al. (2020)	Mixed-methods	*N* = 98. Domestic violence shelter workers (domestic violence/client/shelter advocates, program managers/directors/coordinators) who serve survivors of domestic violence.	(a) Unknown: Domestic violence shelters.	Workers reported compassion fatigue, symptoms of secondary trauma stress.
Choi (2011)	Quantitative survey	*N* = 154. Social workers who serve survivors of family violence, sexual assault, IPV.	(a) Unknown: Agency.	Lower levels of secondary traumatic stress were demonstrated with: (a) greater co-worker, supervisor, and team support; and (b) more access to agency’s strategic information and open communication about agency’s mission and goals.
Colombini et al. (2013)	Qualitative interviews	*N* = 54. Hospital health care providers who serve survivors of IPV.	(a) Government-based: Public hospitals.	Workers experienced feelings of depression, anxiety, inadequacy, helplessness, and frustration because of their inability to solve clients’ problems.
Garcia et al. (2022)	Qualitative interviews	*N* = 53. IPV advocates who serve survivors of IPV.	(a) Unknown: IPV agency.	(a) Workers experienced challenges from working at home during the COVID-19 pandemic. (b) In-person supports were important for debriefing. (c) Workplace flexibility facilitated self-care time and workers’ well-being.
Garimella et al. (2002)	Quantitative survey	*N* = 76. Physicians who serve female survivors of IPV.	(a) Unknown: Hospitals.	(a) Physicians felt that assisting IPV survivors was difficult and stressful. (b) Only 11% of physicians reported positive feelings about their work.
Goldblatt et al. (2009)	Qualitative interviews	*N* = 14. Female social workers who serve survivors and perpetrators of IPV.	(a) Unknown Domestic violence treatment centers, shelters for abused women, telephone hotlines.	Suggested that female workers self-identified with abused women, which resulted in their own intimate relationship conflicts.
Groggel (2023)	Qualitative interviews	*N* = 27. Paralegals, attorneys, law student volunteers, victim advocates who serve survivors of IPV seeking legal services.	(a) Non-profit community service providing legal assistance.	Workers experienced intrusive thoughts of their clients’ traumatic cases, described feeling “slimed” by their traumatic narratives, and mirrored symptoms of secondary trauma.
Jirek (2020)	Qualitative interviews	*N* = 29. Domestic violence and sexual assault advocates, program coordinators, and supervisors who serve survivors of domestic violence and sexual assault.	(a) Non-profit community domestic violence and sexual assault service.	(a) Workers experienced symptoms of secondary traumatic stress and vicarious trauma. (b) They felt stressed and a decreased sense of well-being. (c) They felt their organization failed to provide adequate staffing numbers, living wages, vacation time, and tools to engage in self-care. (d) They felt their workload was unreasonable and the organization had high turnover rates.
Kulkarni et al. (2013)	Quantitative survey	*N* = 236. Domestic violence service providers (legal advocates/case managers, counselors, shelter workers, outreach educators) who serve survivors of domestic violence.	(a) Unknown: Domestic violence agency.	(a) Workers’ compassion satisfaction was associated with longer tenure in the field and shared values with their organization. (b) The strongest risk factor for workers’ secondary traumatic stress and burnout was having unreasonable workloads. (c) Workers’ time spent in leisure had a positive relationship with secondary traumatic stress and burnout.
Laisser et al. (2009)	Qualitative interviews	*N* = 16. Health care workers who serve survivors of IPV.	(a) One government-based hospital. (b) For-profit private service: One privately run hospital.	(a) Workers described feeling frustrated and powerless with limited resources and heavy workloads. (b) They felt degraded by men. (c) They felt a loss of strength and sometimes cried from cases.
Maquibar et al. (2022)	Qualitative interviews	*N* = 19. Primary health care nurses caring for women experiencing IPV.	(a) Government-based: Public health care setting.	(a) Caring for IPV survivors was an emotional burden and sometimes negatively influenced personal lives. (b) Nurses felt fulfilled if survivors left abusive situations.
Pfitzner et al. (2022)	Mixed-methods	*N* = 166. IPV practitioners and service providers who supported women survivors of IPV.	(a) Unknown: Authors did not collect information on specific organizations.	(a) Working from home can be emotionally difficult and disrupt personal safe space and self-care rituals. (b) In-person colleague support, guidance, and debriefing are important for emotional well-being.
Slattery and Goodman (2009)	Quantitative survey	*N* = 148. Domestic violence advocates who supported survivors of domestic violence.	(a) Government-based: Courts. Unknown: Other legal settings, community health centers, crisis and counseling centers, hospitals, social services, domestic violence agencies.	Co-worker support, quality clinical supervision, and respectful workplace environments with shared power were important for emotional well-being and secondary traumatic stress protection.
Van der Wath et al. (2013)	Qualitative interviews	*N* = 11. Hospital emergency nurses who serve survivors of IPV.	(a) Government-based: Two public hospitals.	Workers experienced emotional distress, disruptive and recurrent memories, and somatic symptoms from their job that made them vulnerable to secondary traumatic stress.
Van der Wath et al. (2016)	Qualitative interviews	*N* = 9. Hospital emergency nurses who serve survivors of IPV.	(a) Government-based: Two public hospitals.	Workers attempted to regulate their emotions, sought emotional and spiritual support, and engaged in leisure-based activities to cope with work.
Voth Schrag et al. (2022)	Quantitative survey	*N* = 520. IPV and sexual assault service providers who serve survivors of IPV and sexual assault.	(a) Government-based: Law enforcement victim services. (b) Unknown: Universities, IPV/sexual assault agencies.	Workplace microaggressions and heavy workloads were associated with compassion fatigue among workers.
Voth Schrag et al. (2023)	Qualitative interviews	*N* = 33. IPV and sexual violence service providers who serve survivors of IPV and sexual violence.	(a) Unknown: IPV/sexual violence service agencies.	(a) Experienced moral distress, and decreased motivation, empathy, and emotional well-being related to the inability to meet professional and ethical expectations during COVID-19. (b) Experienced difficulties disconnecting from work, frustration, isolation, and disrupted agency communication from working remotely.
Wachter et al. (2020)	Quantitative survey	*N* = 623. IPV and sexual assault providers who serve survivors of IPV and sexual assault.	(a) Government-based: Legal settings. (b) Non-profit community social service. (c) Unknown: University, IPV/sexual assault, medical settings.	Workers who engaged more frequently in coping behaviors such as leisure-based activities and vacation time reported higher levels of compassion satisfaction.
Watson et al. (2017)	Qualitative interviews	*N* = 17. Therapists serving older women IPV survivors.	(a) Unknown: Primary care mental health services.	Workers experienced helplessness, burnout, and doubted their ability to work meaningfully with clients.
Williams et al. (2021)	Qualitative interviews	*N* = 18. Professionals who care for and serve IPV survivors.	(a) Unknown: Healthcare, legal aid, housing, and social services.	Workers felt overworked, overwhelmed, exhausted, and underpaid.
Wilson et al. (2018)	Qualitative focus groups	*N* = 23. Mental health therapists who serve patients at risk for IPV and suicide.	(a) Unknown: Mental health center.	Workers experienced burnout which contributed to feelings of self-doubt and hindered work productivity.
Wilson et al. (2021)	Qualitative focus groups	*N* = 23. Mental health therapists who serve patients at risk for IPV and suicide.	(a) Unknown: Mental health center.	(a) Workers felt helpless, hopeless, and inadequate in their role. (b) They found it difficult to maintain empathy and patience for survivors who returned to their abusive partner, and felt frustrated at their inability to empower clients. (c) A lack of appropriate training in IPV-specific contexts was reported.
Wood et al. (2020)	Mixed-methods	*N* = 530. IPV and sexual assault support workers who serve survivors of IPV and sexual assault.	(a) Unknown: Dual family violence-sexual assault agency, domestic violence-focused agency, sexual assault-focused agency.	(a) Workers experienced stress and challenges in supporting survivors due to the COVID-19 pandemic. (b) Increased funding, mental health support, and additional resources for victim services were suggestions offered by workers to reduce stress.
Wood et al. (2019)	Quantitative survey	*N* = 352. IPV and sexual assault support workers who serve survivors of IPV and sexual assault.	(a) Government-based: Legal settings. (b) Non-profit community social service. (c) Unknown: College/university, IPV/sexual assault, medical settings.	Low salaries, lack of quality supervision, and high burnout scores were factors associated with lower job satisfaction and turnover intentions.

*Note.* IPV = intimate partner violence.

### Theme 1: Diversity of IPV Workplace Settings and Roles

Across studies, a large proportion failed to clarify the context or setting in which the IPV support workers were employed. The workplace context relates to organizational structure, funding, and supports as these will differ and may or may not influence the health, wellness, and coping strategies of IPV support workers. Also, the defining of credentials and roles of IPV support workers were sometimes difficult to discern. In each country, there will be differences in how IPV services are organized within government-based institutions and in the community. Likewise, the qualifications, training, or credentials required to assume these roles may be quite diverse; professional background and education may be a requirement, yet in some cases, individuals who have personally experienced IPV or trauma may serve as advocates to take on supportive roles (Bemiller & Williams, 2011; Groggel, 2023; Kulkarni et al., 2013). Workplace settings and roles of IPV support workers were common sub-themes to contextualize experiences.

#### Workplace Settings

Four broad structures of workplace settings in which IPV support workers are employed will be presented. The first category is government-based settings, where IPV support workers are employed in structured systems, institutions, or establishments that are provided with consistent public funding, mandates set by government policy, and often access to employee health resources. The settings included: law enforcement agencies and judicial courts (Bell, 2003; Voth Schrag et al., 2022; Wachter et al., 2020; Wood et al., 2019), service bureaus (Ben-Porat, 2017), and in some contexts where socialized medicine or publicly funded healthcare is available, public hospital and healthcare institutions (Colombini et al., 2013; Laisser et al., 2009; Van der Wath et al., 2013, 2016).

The second category where IPV support workers may be employed include non-profit, community-based settings organizations or agencies that are independent, non-profit, not publicly funded, and not bound by government policy (Brend & MacIntosh, 2021; Groggel, 2023; Jirek, 2020; Wachter et al., 2020; Wood et al., 2019). Typically, these organizations encompass emergency shelters, crisis hotlines, victim advocacy and support services, women’s services or women’s aid, and community outreach agencies (Jirek, 2020). Evidence suggests that access to supportive resources and education for workers’ wellness may not always be available in community-based settings due to limited funding (Jirek, 2020). A lack of supportive health and wellness resources, organizational policies, and programs available from the workplace may contribute to challenges in coping with IPV work-related stressors.

A third category included for-profit private service settings. Some studies reported that participants worked in for-profit private therapy practices (Bell, 2003) or privately run hospitals (Laisser et al., 2009). In these contexts, supportive structures may be readily available to IPV support workers depending on revenue generated.

The fourth IPV workplace setting was labeled as unknown representing the largest proportion of studies where clarity and detail of organizational contexts were vague, unclear, or not examined in the study. For instance, Alani and Stroink (2015) reported that participants worked in a range of different organizations, but did not expand on the context of these organizations and if they were government-based, non-profit, or for-profit private. Other studies reported the workplace settings as domestic violence shelters, shelters for violence against women, women shelters, IPV agencies, hotlines, domestic violence coalitions, or centers for prevention of violence. However, lack of detail made it difficult to determine in which domain these were represented. Likewise, in several studies, it was unclear if the healthcare, college, or university settings that IPV support workers were employed in were government-based or for-profit private institutions.

#### Role Qualifications

A common theme from the review relates to diverse roles given that the required credentials, education, and training varied across workplace settings. IPV support workers employed in government-based settings may include nurses, physicians, emergency medical first responders, police officers, judges, or lawyers (Briones-Vozmediano et al., 2014; Colombini et al., 2013; Laisser et al., 2009; Van der Wath et al., 2013, 2016). In these roles, contact with survivors may be limited as exposure to trauma is often short-term during acute crisis; for instance, public hospital workers may occasionally be presented with a patient who experienced IPV.

In non-profit community-based settings, workers may be described generally as victim advocates (Groggel, 2023), domestic violence and sexual assault advocates (Jirek, 2020), or IPV social service providers (Wachter et al., 2020). Counselors, therapists, and social workers may work in either government-based settings, such as social service bureaus (Ben-Porat, 2017), or non-profit, community-based workplaces that serve survivors impacted by IPV (Brend & MacIntosh, 2021). Educational background of workers employed in each of these settings appear to vary greatly and the credentials or skills are inconsistent across the literature; workers’ education ranged from high school, college, Bachelor’s, professional, and graduate degrees (Brend & MacIntosh, 2021; Groggel, 2023; Jirek, 2020), and sometimes, these workers held various roles within their workplace (Brend & MacIntosh, 2021).

Supporting IPV survivors tends to be the workers’ singular focus in for-profit private and non-profit community-based settings and support is often provided over an extended period of time by providing emotional, instrumental, informational, and appraisal support. In their role providing emotional support, they facilitate crisis hotlines using non-judgmental listening; validate survivors’ experiences; utilize psychological therapeutic interventions such as counseling and therapy sessions; offer distress management and mobilize individual coping skills; and assist to build survivor empowerment by reinforcing women’s rights and ability to make their own choices (Bell, 2003; Kulkarni et al., 2013; Wood et al., 2019). Instrumental support is provided through: (a) access to emergency shelters and transitional housing; (b) economic assistance; (c) advocacy and outreach; and (d) accompaniment and support to navigate the health care, legal, and child protection systems (Bell, 2003; Kulkarni et al., 2013; Voth Schrag et al., 2022; Wood et al., 2019). Informational support is facilitated by: (a) individual safety planning and referrals; (b) information about options for survivors’ future; (c) information regarding survivors’ legal rights; and (d) raising public awareness and education on IPV and services offered (Groggel, 2023; Kulkarni et al., 2013). Further, these workers provide appraisal support through the provision of information for client self-evaluation, such as assisting clients in understanding their victimization and that they are not responsible for their own abuse (Kulkarni et al., 2013).

Despite the diversity of IPV workplace structures, funding, supports, and roles, there are commonalities of experiences in relation to health and wellness across IPV workplace settings. The next three themes analyze these commonalities across workplace settings to gain insights on the overall health, wellness, and coping strategies among IPV support workers.

### Theme 2: Meaningful Aspects of IPV Support Work

Supporting survivors of IPV can provide altruistic benefits based on individual worker’s perceptions of experiences and may be important to self-worth and self-esteem. The following sub-themes reflect aspects of work that were meaningful to IPV support workers.

#### Purpose and Fulfillment

Only two studies described the satisfying aspects of working in the IPV field, such as purpose and fulfillment, and while Maquibar et al.’s (2022) sample included nurses from a public health care setting, it was unclear if the IPV support workers in Alani and Stroink’s (2015) study were employed in non-profit, community-based settings. Alani and Stroink (2015) conducted a qualitative study in Canada that explored the well-being of seven sexual and domestic violence support workers and highlighted that some support workers expressed a sense of purpose and fulfillment from supporting their clients.

#### Compassion Satisfaction

Kulkarni et al. (2013) surveyed a large sample of 236 domestic violence service workers to examine factors that contributed to their compassion satisfaction. The term “compassion satisfaction” encompasses feelings of happiness, invigoration, and the sense of satisfaction from assisting others (Kulkarni et al., 2013). The researchers found that workers’ compassion satisfaction was associated with having a longer tenure in the field as well as shared values with their organization (Kulkarni et al., 2013). Although the domestic violence service workers were recruited from domestic violence agencies, the types of workplace settings (i.e., for-profit/non-profit or government-based settings) in the study were unknown.

#### Rewarding and Valuable Work

Bell’s (2003) qualitative study explored the experiences of 30 workers who counseled battered women in the United States. Although the sample included workers employed in for-profit private and government-based settings such as law enforcement agencies, courts, and for-profit private therapy practices, it was unclear if the shelters and hotlines were non-profit, government, or for-profit private settings. The study provided insights on how workers felt that their job was rewarding and satisfying, and the workers expressed feeling more grateful, blessed, less-judgmental, self-worthy, valuable, and compassionate from having the opportunity to be part of their clients’ recovery process.

### Theme 3: Adverse Experiences

Ongoing exposure to the outcomes of abuse, particularly among women, can affect IPV support workers in ways that may be innocuous or harmful to health, wellness, and coping ability. The following sub-themes consider the psychological, physical, and social difficulties that IPV support workers have encountered.

#### Psychological Distress and Physiological Discomfort

A considerable amount of literature discusses the stressors of working with survivors experiencing IPV among hospital workers (e.g., physicians, nurses), and IPV advocates, most of whom were employed in government-based settings. Several studies reported psychological consequences and distressing emotional responses from IPV support work. Workers described caring for IPV survivors as depressing, anxiety-inducing, giving rise to a feeling of helplessness, frustrating, difficult, stressful, and an emotional burden (Colombini et al., 2013; Garimella et al., 2002; Laisser et al., 2009; Maquibar et al., 2022; Van der Wath et al., 2013). The rationale for these feelings was related to the inability to solve clients’ problems and influence their decision to leave an abusive relationship (Colombini et al., 2013), or the inability to maintain intense emotional reactions after the IPV homicide of an individual (AbiNader et al., 2020). A sense of frustration was experienced because clients returned to their abuser, did not report the abuse to the police, did not follow up with referrals, or did not disclose the truth, which made it difficult for them to address their clients’ problems. Likewise, Laisser et al.’s (2009) study explored hospital workers’ perspectives who described feelings of frustration, powerlessness, dejection, and hatred when supporting patients who were experiencing IPV. The feelings of frustration and powerlessness emerged from having little to offer patients when they needed help and expressed a “why bother?” (p.73) attitude when struggling to manage heavy workloads with limited resources (Laisser et al., 2009). As well, they reported: (a) feeling miserable and uneasy from the overwhelming hatred toward perpetrators; (b) feeling degraded by men after witnessing abuse from a husband; and (c), feeling a loss of strength, and sometimes even cried from managing IPV cases (Laisser et al., 2009).

Psychological and physiological discomfort also emerged among emergency nurses who were exposed to the suffering and vulnerability of IPV survivors (Van der Wath et al., 2013). For instance, they experienced emotional distress and disruptive, recurrent memories, making them vulnerable to symptoms of secondary traumatic stress, sadness, depression, shock, fear, and anger toward the perpetrator. One emergency nurse described these emotional experiences as “very much stressing, very much depressing. . .sometimes I used to cry alone” (p. 2246) while others explained how their frequent feelings of sadness intruded on their relationships with families as they could not give them their full attention (Van der Wath et al., 2013). Their recurrent memories were sometimes triggered unexpectedly, and were depicted as “disruptive and vivid mental images, accompanied by strong emotional experiences” (p. 2246) such as visualizing mental images of a man beating up a powerless woman (Van der Wath et al., 2013). Some nurses described somatic symptoms such as feeling “a pressure on [their] chest” (p. 2247) from concern for their IPV client (Van der Wath et al., 2013). Likewise, somatic symptoms of stress such as nightmares, chest pains, and stress-related dizziness were reported among IPV workers from various workplace settings in Bell’s (2003) study.

#### Interpersonal Social Challenges

IPV support workers’ exposure to their clients’ traumatic stories resulted in them feeling more negative toward others, causing disruptions in their own intimate relationships by rejecting their partner’s sexual advances (Bell, 2003). A phenomenological study also highlighted the social aspects of working with IPV survivors among female social workers employed in unknown workplace settings (Goldblatt et al., 2009). The workers’ professional and private lives were blurred and associated with relationship conflicts, divorce, and intimate partnership reassessments. One worker indicated that physical intimacy with their partner was impossible after involvement with a physically abused client (Goldblatt et al., 2009). Notably, researchers suggested that such spillover between professional and private lives may be a result of female workers self-identifying with abused women and an on-going self-examination of their own partnerships. Not all women experienced challenges as one participant reflected on feeling fortunate to have a stable, respectful, and long-lasting relationship with her partner (Goldblatt et al., 2009).

#### Environmental and Organizational Challenges

Numerous studies have described workers’ experiences of low job satisfaction, high turnover intentions, occupational stress, workplace frustrations, and challenging working conditions in various IPV workplace settings. Wood et al. (2019) revealed a significant association between IPV and sexual assault workers and job turnover intentions from American government-based, non-profit community-based and unknown workplace settings. Lower salaries, lack of quality supervision, and high burnout were factors associated with lower job satisfaction and turnover intentions. Although the authors indicated that the sample included employees from legal settings and non-profit community social services, it is unclear which workplace domains they were employed in (Wood et al., 2019).

Occupational stress, moral distress, and decreased emotional well-being was experienced among workers (e.g., counselors, therapists, case managers, service providers, etc.) from unknown IPV and sexual assault agencies during the COVID-19 pandemic (Voth Schrag et al., 2023; Wood et al., 2020). Nearly 95% of workers in Wood et al.’s (2020) study reported feeling a little bit of stress before the pandemic, and over 84% revealed some increase in stress since the pandemic began. Stress was related to transitioning services online due to the pandemic; the workers struggled to establish safety with clients over Zoom and feared that their interaction would be interrupted by the perpetrator (Wood et al., 2020). Reduced access to coping strategies were also reported, for example, a worker stated that “a huge part of my self-care at work was chatting with my coworkers after a hard session” (Wood et al., 2020, p. 10). Increased funding, mental health support, and additional resources for victim services were suggestions offered by these workers to reduce stress. Similarly, studies revealed challenges from working at home during the COVID-19 pandemic among IPV advocates and practitioners from unknown agencies, as they experienced difficulties disconnecting from work and reaching out to colleagues and peers when needing to debrief (Garcia et al., 2022; Pfitzner et al., 2022; Voth Schrag et al., 2023). These findings reflect additional knowledge of the stressors of working during unprecedented times, including the importance of in-person peer supports to debrief after difficult client interactions.

IPV and sexual assault support workers employed in a range of organizations experienced feeling a lack of control and ability to effectively assist their clients (Alani & Stroink, 2015). For instance, they felt that their organization’s policies were limiting, such as the time restrictions for offering services and inability to learn about their clients’ cultural practices. Workers reported how a lack of community resources outside of their organizations made it difficult to support their clients, such as the short-term nature of funding, or the lack of information on what was available for clients. They shared how their clients’ experiences of racism and systematic oppression, as well as the lack of accountability on men for their violent behavior, made their jobs frustrating. The workers worried about their clients, and one described feeling “a hauntingness, or a worry” (Alani & Stroink, 2015, p. 366) as to whether they could have done more to assist their clients. As such, organizational issues may compound stress for IPV support workers.

Staff shortages, increased workloads, funding cutbacks, and reduced salaries hampered IPV support workers’ (e.g., social workers, psychologists, law-enforcement officials, and health professionals) ability to provide effective services in public institutions, non-governmental organizations, and specialized services, and this made them feel under-appreciated in their work (Briones-Vozmediano et al., 2014). Similar experiences of feeling overworked, overwhelmed, exhausted, and being underpaid were also reported among IPV support workers from unknown workplace settings in a recent qualitative study by Williams et al. (2021).

#### Burnout

Numerous studies found that workers in the IPV field demonstrated high levels of burnout, which is often characterized by cynicism, helplessness, emotional exhaustion, depression, and difficulty in effectively doing one’s job (Alani & Stroink, 2015; Bemiller & Williams, 2011; Kulkarni et al., 2013; Watson et al., 2017; Wilson et al., 2018, 2021; Wood et al., 2019). Although it was unclear if the psychological therapists worked in a government, for-profit private or non-profit community-based mental health care setting, participants in Watson et al.’s (2017) study reported feeling a “core state of helplessness” (p. 222) while working with older women survivors of IPV, resulting in burnout. The workers doubted their ability to work meaningfully with their clients; they explained that trying to balance therapeutic work, managing clients that present with a range of violations, and accepting that their clients will likely stay in the abusive relationship resulted in a loss of confidence in their ability to work effectively. In order to avoid these feelings of helplessness, workers avoided asking questions about IPV, they made assumptions of how their clients interpreted their own experiences, and focused on addressing their symptoms rather than the root cause of their symptoms. Symptoms of burnout such as emotional distress, hopelessness, and feelings of self-doubt that hindered their work productivity were also reported by those who worked with survivors of IPV in unknown mental health centers (Wilson et al., 2018, 2021).

A lack of appropriate training in IPV-specific contexts was reported, even though the majority of workers held a graduate degree in marriage and family therapy or mental health counseling (Wilson et al., 2021). Given that the main foci of these studies were to understand worker’s perceived readiness and barriers to addressing IPV, findings are valuable in understanding psychosocial and emotional stressors that lead to burnout.

#### Compassion Fatigue and Secondary Trauma

Similar to the symptoms of burnout, “compassion fatigue” is a term used to describe the gradual feelings of helplessness, hopeless, or disempowerment, which in turn affects the workers’ emotional ability to attend to their clients’ needs (Brown et al., 2020; Voth Schrag et al., 2022). Voth Schrag et al. (2022) conducted a cross-sectional survey to examine risk factors for compassion fatigue among IPV and sexual assault service workers in government-based law enforcement services and other unknown workplace settings. The authors found that recent stressors, higher workloads, and having a job that included direct work time with clients experiencing IPV were associated with higher levels of compassion fatigue. Notably, nearly one-third of workers were exposed to workplace microaggressions related to race, sexism, sexual orientation, gender identity, and disability, which was associated with increased levels of compassion fatigue (Voth Schrag et al., 2022). This reflects another dimension of IPV support workers’ experiences in that discrimination in the workplace may compound the stressors of working with survivors of IPV.

In addition to the concept of compassion fatigue, Brown et al. (2020) highlighted experiences of secondary traumatic stress among domestic violence workers in shelters, but it is unclear how these shelters are funded or mandated. “Secondary traumatic stress” is a term that describes a type of work-induced post-traumatic stress disorder; symptoms can be sudden and often are characterized by avoidance, intrusive thoughts, hyperarousal, and an altered sense of self that can ultimately affect workers’ ability to provide quality support (Brown et al., 2020; Kulkarni et al., 2013; Van der Wath et al., 2013). In Brown et al.’s (2020) study, the findings revealed that 36% of shelter workers reported compassion fatigue, 39% expressed symptoms of secondary traumatic stress, and slightly more than half (51%) felt frustrated by their work (Brown et al., 2020). Ben-Porat’s (2015) study revealed that high levels of secondary trauma resulted in a lower sense of post-traumatic growth among domestic violence therapists; however, the workers’ experiences in these two studies may also have been influenced by supporting child survivors or survivors of violence within a household. In Bell’s (2003) study, approximately 30% of workers felt great emotional distress from assisting battered women and experienced symptoms of secondary traumatic stress; one worker explained how her “perception of the world is forever changed” (Bell, 2003, p. 516) and she viewed the world as an unsafe place of devastation, terror, and human suffering. Similarly, Groggel (2023) reported that paralegals and attorneys in non-profit community-based IPV settings experienced intrusive thoughts of their clients’ traumatic cases and described feeling “slimed by clients’ traumatic narratives or by co-workers’ accounts of difficult cases” (p. 67). In another study, social workers employed in government-based social service bureaus, and unknown shelters and prevention of family violence centers reported symptoms characterized by secondary traumatization, such as despair and helplessness that may undermine workers’ confidence in their ability to function in their job and effectively support clients (Ben-Porat, 2017). Higher levels of managerial support contributed significantly to higher levels of social workers’ sense of role competence. Perhaps this is because managerial support often affects workers’ performance through support, training, and resources that are required to do their job. The findings from Ben-Porat’s (2017) study sheds light on the importance of organizational support to cope with work that may lead to secondary traumatization.

Domestic violence and sexual assault workers employed in a non-profit, community-based organization were ineffective in coping responses and in turn, experienced trauma-related distress (Jirek, 2020). The majority (93%) of workers experienced symptoms of secondary traumatic stress and vicarious trauma such as: headaches, pain, physical and emotional exhaustion, emotional numbness, nightmares, anxiety, feeling socially disconnected from others, and a cessation of hobbies (Jirek, 2020). Staff felt overburdened and overstressed with work; some claimed how “self-sacrifice and exhaustion were viewed as badges of honor” (Jirek, 2020, p. 218) and that the amount of work was unreasonable. One worker felt “that the organization prioritized the well-being of its clients over the well-being of its employees” (Jirek, 2020, p. 218) and felt guilty to request time off work. The workers reported that the organization failed to provide the necessary resources and tools for them to engage in self-care. Because the organization failed to employ adequate staffing numbers, it was difficult for workers to use their accrued paid time off benefits to take a vacation and engage in adequate self-care. The organization also failed to provide workers with a living wage, leading to workers’ increased levels of stress, decreased sense of well-being, and contributed to the organization’s high turnover rate (Jirek, 2020). Similarly, Kulkarni et al. (2013) found that the strongest risk factor for secondary traumatic stress and burnout among domestic violence service workers was having unreasonable workloads. Recognizing the factors that lead to secondary trauma highlights the intersections between the work with survivors and individual level factors; it also demonstrates the challenges of working within non-supportive organizations, as structural forces are not within their control.

### Theme 4: Coping Strategies

Engaging in effective coping strategies can assist IPV support workers in combating work-related stressors and managing the psychological, physical, and social difficulties they encounter. The following sub-themes describe IPV support workers’ strategies to cope with adverse work experiences.

#### Coping Behaviors and Self-care

IPV and sexual assault support workers in legal, non-profit community, and unknown settings engaged more frequently in various coping behaviors such as having time off work, time with family, watching television, and engaging in hobbies and reported high levels of compassion satisfaction (Wachter et al., 2020). Likewise, self-care strategies reported by emergency nurses and sexual/domestic violence support workers included spirituality connections, venting to colleagues and maintaining regular social support, eating well, physical activity, going outside, and listening to music (Alani & Stroink, 2015; Van der Wath et al., 2016). Kulkarni et al.’s (2013) cross-sectional study with domestic violence service workers revealed that time spent at leisure had a positive relationship with secondary traumatic stress and burnout, and time spent in self-care did not have a significant relationship to secondary traumatic stress and burnout. This suggests that spending more time engaging in leisure activities or self-care may not always be a protective mechanism from symptoms of secondary traumatic stress and burnout.

#### Workplace Support and Accommodations

An IPV agency’s responsiveness to workers’ needs were helpful, particularly when they accommodated sick time during the COVID-19 pandemic, adjusted schedules to allow time for self-care and family commitments, and created socialization opportunities (Garcia et al., 2022). Similarly, workplace social support was found to be helpful in mitigating secondary traumatic stress among social workers and domestic violence advocates in several studies (Choi, 2011; Brend & MacIntosh, 2021; Slattery & Goodman, 2009). Social workers in non-profit IPV community services identified five prerequisites for effective workplace social support: (a) the need to feel safe in having work-related conversations without reprisal; (b) feel cared for and that their well-being mattered; (c) have available and trustworthy support; (d) have non-judgmental interactions; and (e) have workplace autonomy that involved having the freedom to manage one’s own schedule and intervention style (Brend & MacIntosh, 2021).

#### Meaningful Sacrifice and Adaptation

Bemiller & Williams (2011)’s study surveyed advocates from unknown domestic violence and sexual assault shelters in the United States and introduced an interesting concept called “good soldiering” to explain how they coped in their work where stress and burnout was a likely outcome. The critical finding of the study was that workers saw themselves as a “good solider,” an identity they possessed that helped alleviate high levels of burnout. “The good solider” is one who continues to fight even under difficult circumstances, and accepts that “meaningful sacrifice is part of the position” (Bemiller & Williams, 2011, p. 104). They acknowledged that their job was stressful with few external rewards, but that adaptation was “necessary to do the work of a good solider” (Bemiller & Williams, 2011, p. 104) and to work in a worthy occupation.

## Discussion

This narrative literature review examined existing qualitative, quantitative, mixed-methods, and review research to understand what is known about the health, wellness, and coping strategies of workers who support survivors of IPV in diverse workplace settings. Individuals’ working life conditions are recognized as a social determinant of health (WHO, 2023), and findings of the present narrative review provide insights on the scope of IPV support workers’ daily work experiences and its influence on their health and wellness. Without clarity of workplace context and the background or training of IPV support workers, it is challenging to pinpoint the individual or structural factors that may directly or indirectly influence workplace stressors or health outcomes in the context of supporting IPV survivors. Irrespective of these gaps in the literature, some common themes were identified across studies in relation to the consequences of supporting IPV survivors. The themes of meaningful and adverse aspects of IPV support work, as well as workers’ coping strategies, provide insight on the experiences of individuals across a spectrum of government-based sectors, for-profit private settings, and non-profit community-based services that provide support to those facing IPV. A few qualitative and quantitative-based studies highlight the meaningful rewards of IPV support work which are important as they provide the fulfillment and motivation to stay in the field; unfortunately, this is not balanced with the adverse effects, and may lead to IPV support workers’ health being compromised and in turn, their decision to leave the field. The literature suggests that engaging in self-care activities, having available supports, adequate training and education, as well as workplace autonomy may mitigate some of the adverse effects of supporting survivors in their daily encounters. Additionally, a novel finding from Bell’s (2003) study suggests that IPV support workers who had a sense of competence about coping, including the capacity to have control and successfully respond to a stressful situation, were less stressed and had lower levels of anxiety and depression. Those who drew upon positive role models, particularly family members, had reduced stress levels because they had someone to compare themselves to and help them frame stressful events as manageable. Further, workers who possessed characteristics such as a positive and hopeful attitude, resiliency, and maintaining good faith, collectively played a role in their coping. Clearly, there are contextual differences related to the type of work, organizational structure, funding, and resources available across the spectrum of IPV workplaces. Non-profit community-based workers who support survivors of IPV engage in many interactions with survivors as their role involves supporting survivors through many stages along their journey of leaving the abusive situation. This entails not only initial contact but also providing emotional, informational, and practical supports while they help survivors to navigate their new life upon entering a rather fragmented complex system of support. For example, survivors will require support for housing, psychological intervention, or legal counsel. Therefore, non-profit community-based workers are more likely to develop long-term relationships with survivors because they will require unique needs to be met as they deal with on-going challenges with new circumstances, and thus, non-profit community-based workers may connect more deeply with their trauma (Rossiter et al., 2020). While some survivors may be successful with the support they receive in creating a new life after abuse or violence, other survivors may return to their previous circumstances due to their inability to manage new role identities, places, and context. As well, the gaps in system and structural services also fail to support women and they have no choice but to return to the abusive situation. Non-profit community-based workers take up the situations of IPV survivors vicariously and may often be discouraged when survivors return to the previous abusive relationships, indirectly assuming responsibility for a failed system. In these cases, non-profit community-based workers may struggle if they do not have the resources to cope, at the individual level with self-care activities or at the system level with organizational supports. Contrarily, IPV support workers that provide short-term support in government-based settings may also experience distress from supporting survivors in the acute situations of crises. However, working in these environments with consistent funding, adequate remuneration (i.e., higher wages and health benefits) and workplace health resources, may amend some of the adverse health-related consequences for these IPV support workers. The way in which IPV support workers manifest work-related experiences and stressors are likely different across diverse settings.

Building resilience of workers may be an end goal of coping with supporting IPV survivors, but it is complex and involves more than simply engaging in self-care activities. The organizations that employ IPV support workers are also responsible for recognizing and valuing the emotional work and health effects that come with supporting survivors in their work. Particularly, among IPV community-based settings, the findings shed light on the limited resources to enhance health and wellness and the diverse nature of organizational structures and supports available (Bemiller & Williams, 2011). Additionally, a report by Rossiter et al. (2020) suggests that IPV support workers employed in American and Canadian community-based settings may not always receive adequate wages and health benefits, or recognition for their work. Struggles of IPV workers are also related to their ability to provide quality services because of limited organizational policies and funding, scarce community resources, and a lack of accessible information for survivors (Alani & Stroink, 2015). By spending concentrated time with IPV survivors and dealing with the restrictions of available resources, IPV workers face unique challenges and in turn, they will need to find ways to cope and maintain health with the individual and structural aspects of their work.

If the goals are to promote resilience, health and wellness, and retain these highly valuable IPV support workers, it would be necessary to know more about system differences that may influence their experiences. The rationale is that leaders in IPV workplaces need to take an upstream approach and intervene before workers experience compassion fatigue or secondary traumatic stress. Physiological, psychological and social stressors of supporting IPV survivors, as well as organizational workplace dynamics that may lead to microaggressions and discrimination or other stressors are also important to consider in the context of IPV workers to guide prevention planning.

The breadth of qualitative studies also presented gaps in that they did not specifically focus on health and wellness, rather they centered on client service provision (Briones-Vozmediano et al., 2014; Brown et al., 2020; Colombini et al., 2013; Williams et al., 2021; Wilson et al., 2018, 2021). However, the studies were valuable in illuminating the complex interconnection of experiences from feeling underappreciated, handling increased workload demands (Briones-Vozmediano et al., 2014; Williams et al., 2021), and experiencing burnout (Colombini et al., 2013; Wilson et al., 2018, 2021), compassion fatigue, and secondary traumatic stress from structural issues (Brown et al., 2020). A better understanding of the interplay between social identities, structural level organizational issues, and environmental workplace challenges will be important to identify ways to reduce stress and create a healthy workplace for workers who support IPV survivors.

### Future Research Directions

Qualitative research is needed to amplify the voices of IPV support workers and provide a holistic and deeper understanding of the contextual differences that exist with working in diverse settings. Future researchers should consider providing more clarity in conducting research that considers context of workplace settings as this may be important in understanding the elements of IPV support workers’ experiences and how it may influence health and wellness. Identifying the work conditions in diverse settings that may lead to workers’ experiences of burnout, compassion fatigue, and secondary trauma is needed to understand what work-related aspects may be influencing their decisions to leave the IPV field. This includes exploring in-depth accounts of structural level issues, discriminatory experiences, and workplace environmental challenges experienced by IPV support workers that may result in harmful health and wellness consequences. Exploring ways in which IPV support work influences workers’ relationships with others is also necessary to better understand how this may interfere in their personal lives and create emotional distress. That said, it would be of value to know what IPV support workers in diverse contexts recommend in terms of resources, support strategies, and structural level changes that would assist them in coping with work-related stressors and to inform the development and/or modification of health promotion interventions. In considering the contributions of the COVID-19 pandemic, future research may be important to examine IPV support workers’ long-term health and wellness consequences in the context of unprecedented circumstances of the pandemic. Future research must consider workers’ level of support or how often they interacted with survivors of IPV as more exposure to survivors’ trauma may affect their work experiences and ability to cope. Particularly, IPV support workers in non-profit community-based settings may require more attention as their capacity with survivors is in a longer phase of recovery and their exposure to secondary trauma may be more extensive. Moreover, the level of education and qualifications required to work in various IPV settings should be explored and clearly articulated in future research as inadequate IPV training or education may in part influence workers’ ability to effectively cope with work-related stressors. Finally, it would be of value for additional research to explore the satisfying aspects of IPV support work in diverse settings as this may be important in understanding what fosters a healthy work environment, and in turn, promote worker retention in the field. Table 2 provides a summary of the critical findings for this study and implications for research, policy, and practice.

**Table 2.** Critical Findings and Implications for Research, Policy, and Practice.

Critical Findings
• This narrative review is unique in that it aims to focus on the experiences of IPV support workers in relation to health, wellness, and coping strategies.
• IPV support workers were commonly found to experience psychological, physiological, and social adverse experiences from their everyday work life in supporting survivors.
• Few studies reported meaningful and satisfying aspects of working in the IPV field.
• Engaging in self-care activities, having available supports, adequate training and education, and workplace autonomy is suggested to assist IPV support workers in coping with work-related stressors.
Implications for Research, Policy, and Practice
• Further research is needed to explore the unique experiences of IPV support workers employed in diverse settings in relation to health and wellness, including how IPV support work influences relationships with others.
• Researchers should consider employing a qualitative design to amplify IPV support workers’ voices and capture in-depth accounts of their perspectives.
• Examining effective coping strategies to foster health and wellness among IPV support workers should be considered for future research.
• IPV organizations and others working in the IPV field should be mindful of the potential psychological, physiological, and social impacts that are commonly experienced by IPV support workers. • Relevant policy-makers in IPV organizations should consider reviewing, modifying, and developing specific organizational policies and programs to provide employee health and wellness resources in this field of work.

*Note.* IPV = intimate partner violence.

## Limitations

The methodological rigor of research was not considered as this is not required in a narrative review (Mays et al., 2005). A comprehensive search strategy was used to identify the available literature on the topic; although, some relevant studies may have been missed. Additionally, although the present review did not focus on workers who support child survivors of violence, some studies included domestic violence workers in their sample, which may involve those who support children or survivors of violence between any members of a household.

## Conclusion

This narrative literature review is unique in exploring what is known about the health, wellness and coping strategies of workers who support survivors of IPV. It has provided insights into IPV support workers’ adverse experiences of supporting survivors that may override the altruistic benefits of their work and lead to harmful health outcomes. The implications are relevant as these adverse experiences can influence IPV support worker retention. As well, health promotion is foundational and important to determine if organization and sector-wide policies need to be reviewed or modified to address the gaps in workplace health in the IPV field. Future research is needed to advance this field of study to examine workers’ role and experiences in diverse settings to learn more about their work, health, and coping strategies as they play a vital role in supporting survivors of IPV.
